# Multi-Omics Analysis of the Gut-Liver Axis Reveals the Mechanism of Liver Injury in Colitis Mice

**DOI:** 10.3389/fimmu.2021.773070

**Published:** 2022-01-06

**Authors:** Luoyi Zhu, Xin Zong, Xiao Xiao, Yuanzhi Cheng, Jie Fu, Zeqing Lu, Mingliang Jin, Fengqin Wang, Yizhen Wang

**Affiliations:** ^1^ National Engineering Laboratory for Feed Safety and Pollution Prevention and Controlling, National Development and Reform Commission, Hangzhou, China; ^2^ Key Laboratory of Molecular Animal Nutrition, Ministry of Education, Zhejiang University, Hangzhou, China; ^3^ Key Laboratory of Animal Nutrition and Feed Science (Eastern of China), Ministry of Agriculture and Rural Affairs, Hangzhou, China; ^4^ Key Laboratory of Animal Feed and Nutrition of Zhejiang Province, Department of Animal Science, Zhejiang University, Hangzhou, China

**Keywords:** IBD, liver injury, gene regulation, gut microbiota, host-microbe interactions

## Abstract

Liver injury is a common complication of inflammatory bowel disease (IBD). However, the mechanisms of liver injury development are not clear in IBD patients. Gut microbiota is thought to be engaged in IBD pathogenesis. Here, by an integrated analysis of host transcriptome and colonic microbiome, we have attempted to reveal the mechanism of liver injury in colitis mice. In this study, dextran sulfate sodium (DSS) -induced mice colitis model was constructed. Liver transcriptome showed significant up- and down-regulation of pathways linked to immune response and lipid metabolism, respectively. Whilst the colon transcriptome exhibited dramatic alterations in immune response and pathways associated with cell growth and death. The microbiota of DSS-treated mice underwent strong transitions. Correlation analyses identified genes associated with liver and colon injury, whose expression was associated with the abundance of liver and gut health-related bacteria. Collectively, the results indicate that the liver injury in colitis mice may be related to the intestinal dysbiosis and host-microbiota interactions. These findings may provide new insights for identifying potential targets for the treatment of IBD and its induced liver injury.

## Introduction

Inflammatory bowel disease (IBD) including ulcerative colitis (UC) and Crohn’s disease (CD) is a chronic intestine inflammatory disease characterized by low mortality and intractable cure (1). Nowadays, IBD has become a worldwide epidemic due to the persistent rise in its incidence, and the burden of IBD poses a challenge to health care systems (2). Of even greater concern is that IBD increases the risk of multiple complications (3, 4). Therefore, reducing the occurrence of IBD will provide a positive effect on human and animal health as well as social development. The onset of IBD is mediated by a variety of factors such as microbes (5), genetics (6), and the external environment (7), etc., however, the exact mechanisms are not yet thoroughly elucidated.

As research on IBD continues, some researchers have observed that patients with IBD frequently suffer from liver disease (8). The development of complications may further aggravate the treatment difficulty and health risk, and yet the underlying molecular mechanisms and therapeutic targets of IBD-induced liver disease are still inadequately characterized. The development of multiple diseases is always closely linked to abnormal gene expression (9). Transcriptomic technologies are widely used in the fields of clinical diagnosis and drug development due to their usefulness in revealing the molecular mechanisms of specific biological processes and disease development through understanding gene function and gene structure (10, 11). Therefore, transcriptome sequencing analysis of liver at risk from colitis is beneficial to uncover the underlying mechanisms of its pathogenesis.

Intestinal dysbiosis often exists in patients with IBD, and the immune response of the intestinal mucosa to dysbiosis exacerbates the dysregulation of intestinal homeostasis (12). The communication of cytokines and inflammatory mediators between the gut and the liver means that dysregulation of gut homeostasis inevitably affects liver health (13, 14). The gut microbiota performs an essential role in maintaining intestinal homeostasis and regulates host gene expression (15–17). Nevertheless, the role of intestinal dysbiosis in gene expression and injury of colon and liver under the context of colitis has not been systematically reported to date.

Herein, Dextran Sulfate Sodium (DSS) -induced colitis model was employed in this study. Comparison of the colonic microbiome (16S rRNA sequencing) and differential gene expression in the colon and liver (RNA-sequencing) of the colitis and healthy group was used to discern the relevant signaling pathways and potential molecular mechanisms that lead to tissue injury. Furthermore, correlations between gut microbes and colonic and liver gene expression were identified. The aim of this study is to unravel the potential mechanisms underlying the development of colitis and its induced liver injury through exploring the potential link between microbes and host genes. These findings will not only expand our knowledge of IBD, but may also provide new avenues for finding potential therapeutic targets for the treatment of IBD and its complications such as liver disease.

## Materials and Methods

### Animal Studies

**Figure 1:**
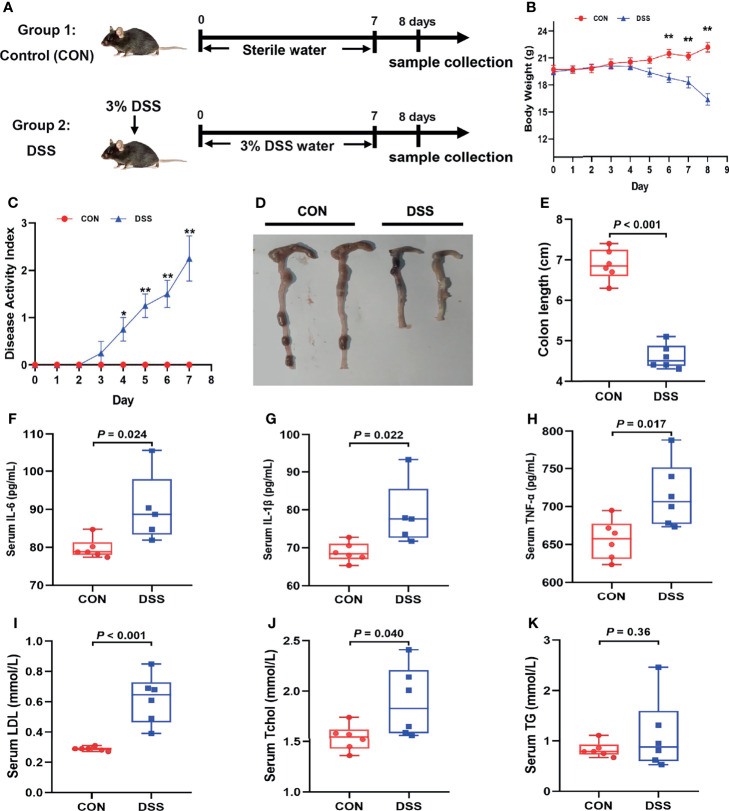
DSS-induced acute colitis, systemic inflammatory response and lipid metabolism disorders in mice. **(A)** Schedule of the experiment treatment; **(B)** Body weight loss; **(C)** Disease activity index score; **(D)** Morphological observations of colon; **(E)** Colon length; **(F–H)** The concentrations of IL-6, IL-1β and TNF-α in serum; **(I–K)** the content of LDL, Tchol and TG in serum. Data were expressed as means ± SEM (n = 5-6/group). *P < 0.05, **P < 0.01.

All animal experiments were approved by the Animal Care and Use Committee and the Animal Experimentation Ethics Committee of Zhejiang University (Hangzhou, China). Twelve 6-week-old C57BL/6 mice were purchased from Shanghai SLAC Laboratory Animal Co. Ltd, and the mice were housed in a specific-pathogen-free (SPF) environment with a temperature of 18- 22°C, 55± 5% humidity, and a 12-hour cycle of light/dark. After one week of acclimatization, the mice were used for the experiment. Each mouse was weighed on day 0 of the experiment and randomly divided into control (CON group) and DSS groups (6 per group). The mice in the DSS group drank sterilized water containing 3% DSS (36- 50 kDa; MP Biomedicals, California, USA) ad libitum for 7 consecutive days, and the control group drank sterilized distilled water. All mice were allowed to feed ad libitum throughout the study. The status of the mice including mental status, diarrhea and stool bleeding were recorded during the experiment and samples were collected at day 8 (**Figure 1A**).

### Disease Activity Index (DAI)

Mice in the CON and DSS groups were scored daily using the DAI. The DAI was assessed in accordance with the standard scoring system previously defined by Wang et al. and Chen et al. (18, 19), which included body weight loss (0 = no loss, 1 = 0.1- 5%, 2 = 5- 10%,3 => 10%), stool consistency (0 = normal, 1 = regular shape but wet, 2 =mild diarrhea, 3 =diarrhea), stool bleeding (0 = no blood, 1 = little blood in stool, 2 =gross blood in the stool, 3 = blood in all stool) and mice condition (0 = normal, 1 = slightly poor condition, 2 =moderate poor condition, 3 = poor condition).

### Histologic Analysis and Histological Evaluation

After the mice were sacrificed, the length of the colon was measured. Approximately 0.5 cm of colon was fixed in 4% formaldehyde for more than 24h and embedded in paraffin, followed by slicing and staining with hematoxylin and eosin. Images of the sections were acquired using a Leica DM3000 microscope (LEICA, Wetzlar, Germany) and the crypt depth were measured using the Image J Software (NIH Image, Bethesda, MD). Histological evaluation was performed as described in previous report (20). Histological score was assessed according to the degree of inflammatory cell infiltration (0-3, from few inflammatory cells in the lamina propria to transmural extension of inflammatory cell infiltration) and tissue damage (0-3, from no mucosal damage to profound structural damage of the intestinal wall).

### Serum Cytokine Levels Analysis

The levels of serum cytokines IL-6, IL-1β and TNF-α were performed as described by the manufacturer’s instructions (Jiangsu Meibiao Biological Technology Co. Ltd., Jiangsu, China) and measured at 450 nm with a MultiskanSky Microplate Reader (Thermo Fisher Scientific, Waltham, USA).

### Detection of Serum Biochemical Parameters

The serum lipid profiles including triglyceride (TG), total cholesterol (TChol) and low-density lipoprotein (LDL) were determined using a Hitachi 7600-020 automatic clinical chemistry analyzer (Hitachi, Tokyo, Japan).

### Transmission Electron Microscopy (TEM)

The tight junctions (TJs) and microvilli in colon were observed by TEM as previously described (21). The colon was first fixed with 2.5% glutaraldehyde in phosphate buffer (0.1 M, pH 7.0) for 24 h and then rinsed 3 times in the phosphate buffer (0.1M, pH7.0) for 15 min each, then postfixed with 1% OsO4 in phosphate buffer for 1h followed by three washes in 0.1 M phosphate buffer (pH 7.0) for 15min at each step. After double fixation, samples were firstly dehydrated by a graded series of ethanol (30%, 50%, 70%, 80%) for about 15min at each step, and then with acetone graded (90%, 95%) for about 15 min each step. Finally, the samples were dehydrated twice with absolute acetone for 20 min each. The specimens were then placed in a 1:1 mixture of absolute acetone and final Spur resin mixture for 1 hour at room temperature, then transferred to a 1:3 mixtures of absolute acetone and final resin mixture for 3h and to final Spur resin mixture overnight. The specimens were heated (70°C) in Spurr resin for more than 9h. Specimens were sectioned in LEICA EM UC7 ultratome (LEICA, Wetzlar, Germany) and then stained with uranyl acetate and basic lead citrate for 5-10 minutes, respectively, and observed in Hitachi H-7650 (Hitachi, Tokyo, Japan).

### RNA Isolation and Quantitative Real Time PCR (qRT-PCR)

The qRT-PCR was performed as described by Zong et al. (22). Total RNA of colon was extracted with Trizol reagent (Invitrogen, CA, USA). The quality of the extracted RNA was evaluated using NanoDrop2000 (Thermo Fisher Scientific, Waltham) and the absorbance rates (260/280 nm) of all samples ranged from 1.8 to 2.0. The first-strand cDNA was obtained by reverse transcription reaction of 1 µg RNA using random primers with a reverse transcription kit (Invitrogen, CA, USA) following the manufacturer’s instructions. Subsequent qRT-PCR was performed on StepOnePlus Real Time PCR system (Applied Biosystems, Foster City, USA) with SYBR Green dye (Roche, Mannheim, Germany) and gene-specific primers (**Supplementary Table 1**). The target genes relative mRNA expression was analyzed by the 2-ΔΔCT method. β-actin housekeeping gene was used as normalization control.

### 16S rRNA Sequencing and Bioinformatics Analyses

The obtained colon contents were snap frozen in liquid nitrogen and stored at -80°C until 16s rRNA sequencing was performed. For 16s rRNA sequencing, first, genomic DNA was extracted from the gut contents according to the QIAamp DNA stool Mini Kit (Qiagen, Hilden, Germany) instructions and then assayed the DNA purity and integrity by NanoDrop2000 (Thermo Fisher Scientific, Waltham) and agarose gel electrophoresis, respectively. Subsequently, the V3-V4 region of 16S rDNA (338F: ACTCCTACGGGAGGCAGCA, 806R: GGACTACHVGGGT-WTCTAAT) was amplified through PCR, and the amplified products were detected by 2% gel electrophoresis and recovered by axyprep DNA gel extraction kit (Axygen, USA). Miseq libraries were then generated on the Illumina MiSeq platform (Illumina, Madison, USA) for pyrosequencing. Clean sequences with ≥97% resemblance were assigned to the same operational taxonomic units (OTUs). The free online cloud platform (https://cloud.majorbio.com/) of Majorbio (Shanghai, China) was used to analyze the data. Briefly, alpha-diversity indices including coverage index, numbers of OUT and Shannon index were analyzed by student’s t-test at OTU level. PCoA analysis was based on weighted unifrac distance algorithm (23). Significant differences in relative abundance at the phylum and genus levels were examined by Kruskal-Wallis rank-sum test. Linear discriminant analysis (LDA) effect size (LEfSe) (24) analysis was performed using the non-parametric factoria Kruskal-Wallis sum-rank test to obtain significantly different species between two groups, followed by Wilcoxon rank sum test between two groups to detect between-group differences, and finally LDA was applied to estimate the effect of each species abundance on the differences. PICRUSt2 (25) was applied to predict the function of the gut microbiota.

### RNA Sequencing and Data Analysis

RNA sequencing analysis was performed on pertaining samples as described by Kong et al. (26). Total amount and integrity of RNA extracted from mouse liver and colon tissues were accurately detected with an Agilent RNA 6000 Nano Kit of the Bioanalyzer 2100 system (Agilent Technologies, CA, USA). For library preparation, double-stranded cDNA derived from total RNA as template was used for PCR amplification, then PCR products were purified using the AMPure XP system and library quality was assessed on the Agilent Bioanalyzer 2100 system. The qualified library preparations were sequenced on an Illumina Novaseq platform. PCA analysis based on gene expression (FPKM) of all samples. Differential expression analysis between the two groups was performed using the DESeq2 (27). To ensure accurate differentially expressed genes (DEGs) results, as previously reported noted (28, 29), genes with an adjusted P-value < 0.05 obtained using the method of Benjamini and Hochberg screened by DESeq2 and Fold change ≥ 2 were considered to be differentially expressed. The clusterProfiler R package was used to perform the DEGs in the KEGG pathway for statistical enrichment. We screened for genes enriched in immune response and lipid metabolism-related pathways by KEGG pathways enrichment analyses for the top 250 DEGs in the colon and liver. Correlations between the colonic microbiota and the screened genes were then revealed by Spearman correlation analysis.

### Statistical Analysis

All outcomes were assessed using a two-tailed Student’s t-test of SPSS version 26.0 (SPSS, Inc., Chicago, IL, USA), P < 0.05 was defined as the significance level, all data are expressed as mean ± SEM. The GraphPad Prism version 8.0 (GraphPad Prism, San Diego, California, USA) and R version 4.0.5 were applied to visualize the data.

## Results

### DSS-Induced Acute Colitis, Systemic Inflammatory Response and Lipid Metabolism Disorders in Mice

DSS is typically applied to elicit the mouse model of colitis because of the strengths of simplicity, reproducibility of induced colonic lesions, and the ability to simulate the clinical and histological features of IBD, especially ulcerative colitis (30, 31). Therefore, to investigate the potential mechanisms of the occurrence of colitis and its adverse effects on the organism, a mouse model of acute colitis was established by supplying mice with sterilized water containing 3% DSS for one week (**Figure 1A**). As shown in **Figures 1B–E**, DSS treated mice exhibited significant body weight loss, dramatic increase in DAI and remarkable shortening of colonic length in comparison with the CON group. Furthermore, serum levels of cytokines as IL-6, IL-1β and TNF-α were significantly heightened in the DSS group (**Figures 1F–H**). Interestingly, serum biochemical parameters results indicated that DSS treatment significantly elevated serum TChol and LDL levels (**Figures 1I, J**), while serum TG showed an ascending trend (**Figure 1K**). Overall, the above results suggested that DSS provoked a gross inflammatory response and lipid metabolism disorders in mice.

### DSS Treatment Induced Liver Injury and Altered Gene Expression in Mice

**Figure 2:**
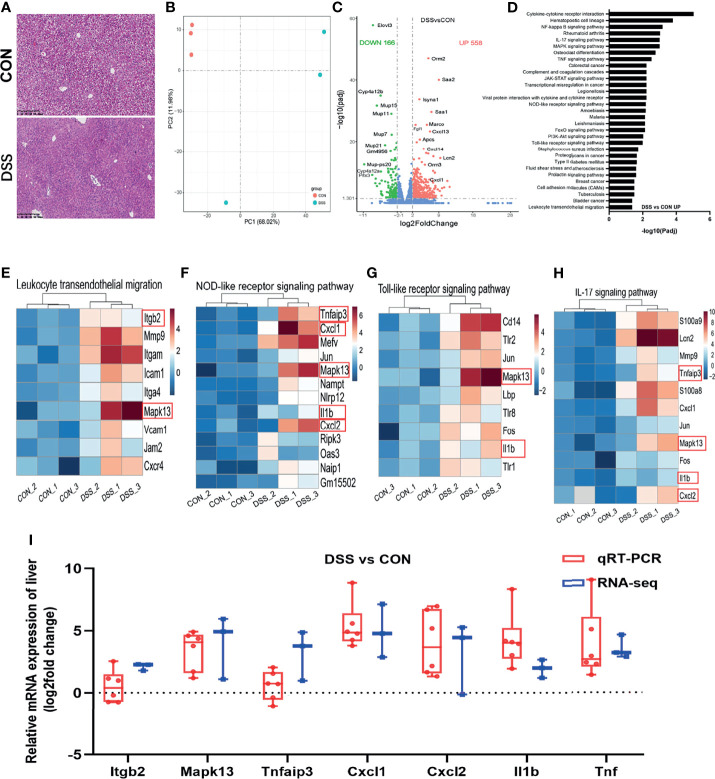
Differentially expressed genes in the liver of healthy (CON) and acute colitis (DSS) mice. **(A)** H&E staining of liver, the bars represent 100μm; **(B)** PCA plot colored by DSS group samples in green and CON groups samples in red; **(C)** Volcano plot showing the changes of liver genes (fold change ≥ 2); **(D)** KEGG functional analysis reveals the biological functions that are enriched in the significantly up-regulated expressed genes; **(E–H)** Heatmap of 4 immune-related pathways enriched in the significantly up-regulated expressed genes; **(I)** qRT-PCR(n=6). confirmations of DEGs screened based on RNA-seq analysis (n=3).

**Figure 3:**
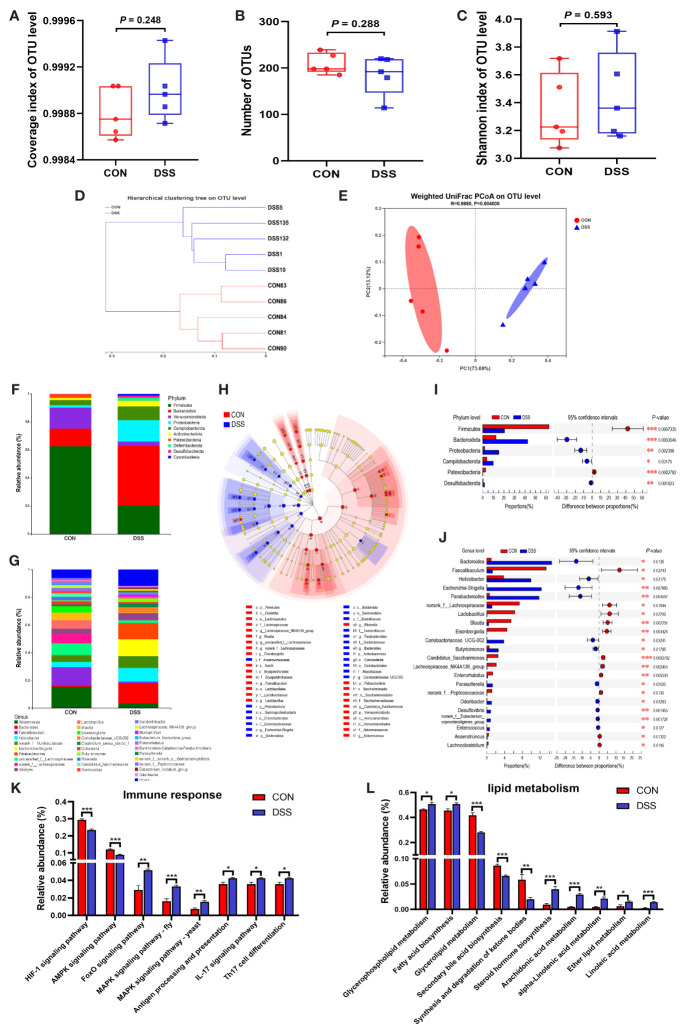
Alterations in the gut microbiota of mice with DSS-induced colitis. **(A)** Coverage index of OUT level; **(B)** Number of OTUs; **(C)** Shannon index of OUT level; **(D, E)** β-diversity was estimated by the hierarchical clustering tree **(D)** *and* PCOA **(E)** on OUT level; **(F, G)** The relative abundance of bacteria at the phylum **(F)** and genus **(G)** levels; **(H)** Cladogram of LEfSe multi-level species difference discriminant analysis (LDA > 4), different color nodes indicate microbial communities that are significantly enriched in the corresponding groups and significantly different between groups; **(I, J)** Comparative analysis of the relative abundance of bacteria at the phylum **(I)** and genus **(J)** levels; **(K, L)** The different abundances of gut microbiota at immune response **(K)** and lipid metabolism **(L)** pathways. Data were expressed as means ± SEM (n = 5). *P < 0.05, **P < 0.01, ***P < 0.001.

**Figure 4:**
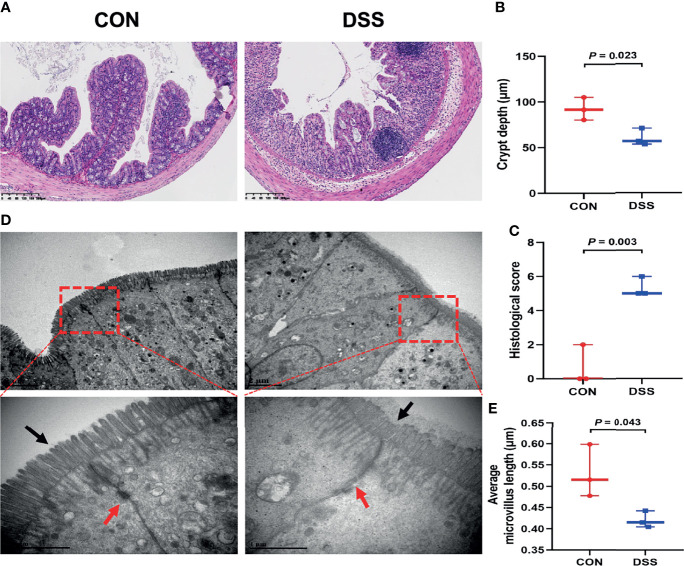
DSS-induced colonic histological changes and barrier dysfunction in mice. **(A)** The representative H&E staining of the colonic sections, the bars represent 200μm; **(B)** The colonic crypt depth; **(C)** histological scores; **(D)** Ultrastructural changes of TJs and microvilli under transmission electron microscopy (12,000× and 40,000×); **(E)** Average microvillus length. Data were expressed as means ± SEM (n = 3).

IBD is considered to be one of the factors affecting liver homeostasis, patients with IBD often present with symptoms of liver injury (8, 32). In this study, increased serum levels of cytokines and lipid metabolism-related indicators in the DSS group indicated impaired liver function (33, 34). Histological analysis of the liver showed swollen hepatocytes, larger nuclei, narrower hepatic sinusoids, and infiltration of inflammatory cells between the hepatic sinusoids and lobules in the DSS group (**Figure 2A**). Thus, liver transcriptome analysis was performed to further elucidate the molecular mechanisms underlying the occurrence of liver injury in a DSS-induced colitis model. The PCA plot revealed a significant difference in the abundance of liver transcripts between the CON and DSS groups, with one sample in the DSS group being somewhat distant from the other two samples (**Figure 2B**). As shown in the volcano plot results in **Figure 2C**, when adjusted *P*-value < 0.05, Fold change ≥ 2 was used as the screening criterion, 166 genes were significantly down-regulated and 558 genes were significantly up-regulated in the DSS group compared with the CON group. KEGG functional clustering analysis of DEGs revealed that DSS treatment led to a significant increase in immune response-related pathways such as “Leukocyte transendothelial migration”, “NOD-like receptor signaling pathway”, “Toll-like receptor signaling pathway” and “IL-17 signaling pathway” compared to the CON group (**Figure 2D** and **Supplementary File 1**). While significant reductions were observed in the pathways involved in lipid metabolism, including “Fatty acid elongation”, “Fatty acid degradation” and “PPAR signaling pathway” (**Supplementary Figure 1A** and **Supplementary File 1**). The heatmaps displayed DEGs enriched in 4 immune response pathways (**Figures 2E–H**) and 3 lipid metabolism-related pathways (**Supplementary Figures 1B–D**). To validate the findings of RNA-seq analysis, a total of 11 DEGs in these pathways were selected for qRT-PCR analysis to further investigate their expression profiles. The expression of 11 genes in the heatmap measured by qRT-PCR demonstrated similar alterations to RNA-Seq analysis, including 7 genes (*Itgb2*, *Mapk13*, *Tnfaip3*, *Cxcl1*, *Cxcl2*, *IL1b* and *Tnf*) with up-regulated expression (**Figure 2I**) and 4 genes (*Cyp4a12b*, *Fabp2*, *Elovl3* and *Elovl5*) with down-regulated expression (**Supplementary Figure 1E**). Of these genes, *Cxcl2* manifested the greatest upregulation and *Elovl3* presented the greatest downregulation. *Cxcl2* has been shown to pose as a gene involved in the immune response (35). *Elovl3* is engaged in maintaining lipid homeostasis by replenishing the intracellular triacylglycerol pool (36).

### Dysbiosis of Gut Microbiota Induced by DSS Treatment

Liver is a major organ exposed to gut-derived substances, numerous studies have proven that liver health strongly associated with the gut homeostasis (14, 37). The gut microbiota plays a vital role in maintaining gut homeostasis to shield us from diseases related to dysbiosis, such as IBD (38). In light of these studies, we further explored the role of gut microbiota in the risk of liver injury in DSS-induced colitis. **Figure 3A** showed that the Coverage index was greater than 0.999 in both the CON and DSS groups, indicating that the sequencing depth reached the requirements for subsequent analysis. A trend of decrease in the number of OTUs was observed in the DSS group compared with the CON group (**Figure 3B**) and there was no significant difference in the diversity of intestinal bacteria as indicated by the Shannon index (**Figure 3C**). The *β* diversity analysis is shown in **Figures 3D, E**. The hierarchical clustering tree outcomes on OTU level revealed that the bacterial structure exhibited significant clustering in both groups (**Figure 3D**). Additionally, the results of principal coordinates analysis (PCoA) based on weighted unifrac metrics (**Figure 3E**) showed that bacterial community structure was distinctly different between the CON and DSS groups, indicating a clear separation between the microbiota groups. Subsequently, the relative abundance of bacteria was further assessed at the phylum and genus levels, respectively (**Figures 3F, G**). At the phylum level, 10 bacteria had a relative abundance of over 1% in at least one group, of which the highest abundance in the CON and DSS groups were Firmicutes and Bacteroidota, respectively, while there were 31 bacteria with relative abundances above 1% at the genus level. Furthermore, significant differences in microbiota structure between the two groups were revealed by cladogram plots of LEfSe analysis (LDA>4) (**Figure 3H**). As shown in **Figures 3I, J**, at the phylum level, the relative abundance of Firmicutes and Patescibacteria was significantly lower in the CON group than in the DSS group based on the Kruskal-Wallis rank sum test, while the relative abundance of Bacteroidota, Proteobacteria, Campilobacterota and Desulfobacterota had significantly higher relative abundance than the DSS group. At the genus level, 17 bacteria were significantly different among those with relative abundance greater than 1%, the relative abundance of harmful bacteria such as *Helicobacter*, *Escherichia-Shigella* and *Coriobacteriaceae_UCG-002* was notably elevated in the DSS group, while the relative abundance of beneficial bacteria as *Faecalibaculum*, *norank_f:Lachnospiraceae* and *Lactobacillus* were significantly decreased. These data supported that DSS treatment gives rise to disorders of the gut microbiota. The function of the intestinal microbiota depends on its composition and abundance. Therefore, PICRUST 2 was employed for metabolic functions prediction of the gut microbiota. The findings revealed that there were significant variations in 5 of the 6 pathways of KEGG level 1, except for no differences in genetic information processing (**Supplementary Figure 2A**). In addition, 24 pathways at KEGG level 2 differed considerably between the two groups (**Supplementary Figure 2B**). At KEGG level 3, we focused on the pathways related to immune response and lipid metabolism. As shown in **Figure 3K**, immune response-related pathways such as MAPK signaling pathway, IL-17 signaling pathway and Th17 cell differentiation were dramatically up-regulated in the DSS group. Pathways related to lipid metabolism like Glycerophospholipid metabolism, Fatty acid biosynthsis were significantly up-regulated in the DSS group, while Glycerolipid metabolism, Secondary bile acid biosynthesis, Synthesis and degradation of ketone bodies were markedly down-regulated (**Figure 3L**). The above results indicated that DSS treatment induced obvious compositional and structural shifts in the colonic microbiota of mice, as well as further influenced the metabolic functions of gut microbiota.

### DSS Treatment Induced Colon Histopathology and Gene Expression Alterations in Mice

**Figure 5:**
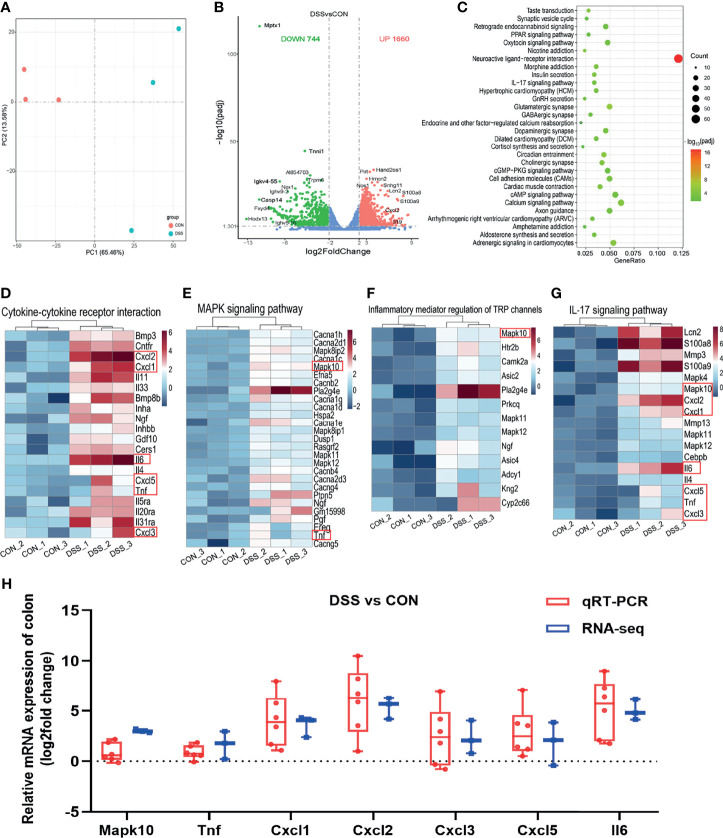
Differentially expressed genes in the colon of healthy (CON) and acute colitis (DSS) mice. **(A)** PCA plot colored by DSS group samples in green and CON groups samples in red; **(B)** Volcano plot showing the changes of liver genes (fold change ≥2); **(C)** KEGG functional analysis reveals the top 30 biological functions that are enriched in the significantly up-regulated expressed genes; **(D–G)** Heatmap of 4 immune-related pathways enriched in the up-regulated expressed genes; **(H)** qRT-PCR (n=6) confirmations of DEGs screened based on RNA-seq analysis (n=3).

As the site of direct exposure to the colonic microbiota, dysbiosis of the gut bacteria is one of the factors contributing to colitis (39). To further investigate the functions of colonic microbiota in the specific biological processes and molecular mechanisms involved in the colitis development, we also performed histological analysis and RNA sequencing of colonic tissues. As illustrated in **Figure 4A**, H & E staining displayed striking evidences of colonic crypt loss and inflammatory cell infiltration in the DSS group, the further statistical findings also indicated shallow colonic crypt depth in mice (**Figure 4B**), and the histological scoring results suggested the colon was grossly damaged (**Figure 4C**). The intestinal microvilli morphology and TJs were observed using TEM (**Figure 4D**). Compared with the CON group, the microvilli length in the DSS group was significantly shorter (**Figure 4E**). TJs are the foremost component in building the intestinal barrier. **Figure 4** demonstrated that TJs got less and disorganized, and the cell borders were blurred. These findings implied that intestinal function was disrupted in DSS-challenged mice and triggered an intestinal inflammatory response. Based on the RNA sequencing from the colon, PCA analysis showed segregation of colonic transcripts abundance in the CON and DSS groups (**Figure 5A**). In terms of gene expression changes, 744 genes were markedly down-regulated and 1660 genes were significantly up-regulated in the DSS group compared with the CON group when adjusted *P*-value < 0.05 and fold change ≥ 2 was used as a screening criterion (**Figure 5B**). Subsequently, KEGG functional clustering analysis of DEGs revealed that treatment with DSS resulted in up-regulation of some pathways associated with immune responses in the colon (**Figure 5C** and **Supplementary File 2**). The heatmap displayed DEGs enriched in four up-regulated immune response pathways such as “Cytokine-cytokine receptor interaction”, “MAPK signaling pathway”, “Inflammatory mediator regulation of TRP channels” and “IL-17 signaling pathway” (**Figures 5D–G**). Notably, pathways down-regulated in the colon were mainly involved in cell growth and death and DNA damage repair (**Supplementary Figures 3A–C** and **Supplementary File 2**). Furthermore, to evaluate the results of RNA-seq analysis, a total of 10 DEGs involved in the pathways were selected for qRT-PCR analysis. qRT-PCR expression findings of these genes were consistent with RNA-Seq analysis (**Figure 5H** and **Supplementary Figure 3D**), including 7 genes with increased expression (*Mapk10*, *Tnf*, *Cxcl1*, *Cxcl2*, *Cxcl3*, *Cxcl5* and *IL6*) and 3 genes with decreased expression (*Mcm6*, *Mcm5* and *Fads2*). Among these genes, *Cxcl2* as a gene with known involvement in the immune response exhibited the greatest up-regulation (35). *Fads2*, a gene reported to be mutation-prone in IBD patients, showed the greatest downregulation in our study (40).

### Correlation of DSS-Induced Gut Microbiota Dysbiosis With DEGs in the Liver and Colon

**Figure 6:**
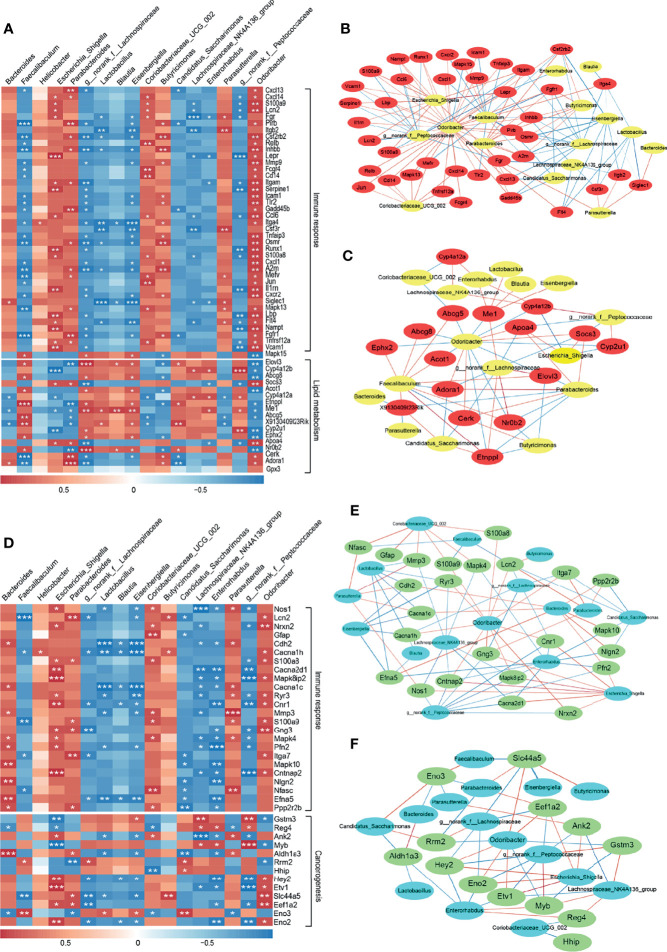
Interaction between gut microbiota and DEGs in the colon and liver in healthy (CON) and acute colitis (DSS). **(A, D)** Correlation of gut microbiota with genes enriched for immune response and lipid metabolism or cancerogenesis-related functions among the top 250 DEGs in the liver **(A)** and colon **(D)** (*P < 0.05, **P < 0.01, ***P < 0.001). **(B, C)** Network visualization of significant gene-microbe correlations in the liver associated with immune response **(B)** and lipid metabolism**(C)** (*P* < 0.05). **(E, F)** Network visualization of significant gene-microbe correlations in the colon associated with immune response **(E)** and cancerogenesis **(F)** (*P* < 0.05). Blue and red edges represent negative (R < -0.85) and positive (R > 0.85) correlations, respectively, respectively. Edge thickness indicates the strength of the correlation.

With the purpose to investigate the microbe-host gene expression relationships and their potential mechanisms in DSS-induced colitis and liver injury in mice. First, we screened 41 and 17 genes associated with immune response and lipid metabolism, respectively, from the top 250 DEGs in liver tissues, and the top 250 DEGs in colon tissue with 24 and 13 genes related to immune response and cancerogenesis, respectively. Then, 17 bacteria with relative abundance greater than 1% at the genus level and with significant differences were selected (**Figure 3J**). On this basis, we performed a Spearman correlation analysis and visualized the correlation between the relative abundance of bacteria and gene expression of liver and colon in **Figures 6A, D**, respectively. Particularly, we observed some significant positive gene-bacterial correlations between *Helicobacter*, *Escherichia-Shigella*, *Coriobacteriaceae_UCG-002*, etc. with immune response-related genes and some significant negative gene-bacterial correlations with lipid metabolism-related genes in liver tissues. However, *Faecalibaculum*, *norank_f:Lachnospiraceae*, *Lactobacillus*, etc. showed opposite trends of association with these genes (**Figure 6A**). In the colon, the correlation between bacterial and immune response-related genes was similar to that in the liver. Interestingly, only a subset of genes related to cancerogenesis showed positive correlations with *Helicobacter*, *Escherichia-Shigella*, *Coriobacteriaceae_UCG-002*, etc. while *Faecalibaculum*, *norank_f:Lachnospiraceae*, *Lactobacillus*, etc. presented negative correlations with these genes (**Figure 6D**). Furthermore, we visualized the results of strong correlations with *P* < 0.05 and R > 0.85 or R < -0.85 using Cytoscape software. As shown in **Figures 6B, C**, *Escherichia_Shigella* was the main microbiota that presented positive correlations with immune response-related genes in the liver, which showing the highest correlation with *Lepr*. *norank_f:Lachnospiraceae*, *Faecalibaculum* and *norank_f:Peptococcaceae* were the main microbiota exhibiting positive correlations with genes linked to lipid metabolism, and the highest positive correlation was found between *norank_f:Lachnospiraceae* and *Elovl3*, *Faecalibaculum* and *Etnppl*. However, the correlation between *Escherichia_Shigella* and *Cyp4a12b* showed the highest negative correlation (**Supplementary File 3**). In the colon, the main microbiota showed positive correlations with immune response-related genes were *Escherichia_Shigella* and *Parasutterella*, of which, *Escherichia_Shigella* had the highest correlation with *Cntnap2*. Moreover, *Escherichia_Shigella* and *g:norank_f:Peptococcaceae* were the main microbiota associated to cancerogenesis -related genes, and *Escherichia_Shigella* was the most negatively associated with *Myb* (**Figures 6E, F** and **Supplementary File 4**). Thus, it was evident that among these representative correlations, changes in the gut microbiota of DSS-treated mice were closely related to the expression of host genes, where the increase of the pathogenic bacterium *Escherichia_Shigella* in the DSS-induced colitis mouse might be an important factor in causing alterations in host gene expression and disease development.

## Discussion

With the global trend of increasing incidence and prevalence of IBD, it demonstrates that IBD has become a global disease (41, 42). DSS is commonly used to induce colitis to study intestinal diseases (43). In this study, after drinking sterilized water containing 3% DSS for one week, mice exhibited visible colitis phenotypes including decreased body weight, increased DAI and shortened colon length, which were consistent with the previous reports (44, 45). Elevated serum levels of cytokines are one of the hallmark features of mice and patients with colitis (46, 47). Similar to previous reports, the elevated serum levels of IL-6, IL-1β and TNF-α in the DSS-treated group indicated the appearance of severe inflammation. Disruption of the intestinal epithelial barrier including histological damage (48) and breakdown of TJ structures (49) is another characteristic of colitis. Our results also showed severe intestinal histological damage and disruption of the structural integrity of TJ in the DSS group, which further indicated that DSS successfully induced colitis. Interestingly, we observed significant variations in serum TChol and LDL, which were consistent with the findings of Rtibi et al. (50) and Zheng et al. (51). In addition, the TJ structural damage could be one of the reasons of elevated serum lipid levels. Nevertheless, it was reported that Tchol and LDL levels were lower in IBD patients compared to healthy subjects, which is contrary to the findings of this study. For TG levels, there were no significant changes in both IBD patients and mice with colitis (52). These results implied that although there were differences in the lipid profiles of DSS-induced colitis mice and IBD patients, colitis was closely associated with disturbed lipid metabolism. It is well known that the liver is a major handler of lipid metabolism (53), and the disturbances in lipid metabolism are one of the signals of liver injury (54). Not surprisingly, hepatocyte necrosis and inflammatory cell infiltration were observed in the liver tissue of the DSS group in this study, which was also reported by Duan et al. (34). Overall, these results further suggested that both the colon and liver are impaired to varying degrees in DSS-induced colitis, such as inflammatory responses and lipid metabolism disorders. Meanwhile, these results revealed differences between DSS-induced colitis and human colitis, implying that this model could only provide valuable information for human colitis study in some aspects such as preliminary mechanistic exploration and as a vehicle for potential biomarker identification.

Liver injury is proven to be a common extra intestinal manifestation of IBD, however, the precise mechanism of its evolution is still unknown (55, 56). Numerous studies have demonstrated the role of host gene regulation in multiple disease pathogenesis (9, 57). To the best of our knowledge, our work is the first comprehensive transcriptome analysis focused on the liver of colitis mice. In this study, the variation in the DSS group as demonstrated by the PCA plot might be related to biological variability such as differences in the sensitivity of mice to DSS, which could not be eliminated by sequencing technology (58). Conesa et al. (28) reported that the statistical power could reach 87% when sequenced at 30 million mapped reads (an average of 41 million mapped reads in current study) and a sample size of 3 with fold change = 2 in a human blood RNA-seq. Therefore, to ensure the reliability of the screened DEGs, fold change = 2 was used for DEGs screening. Next, we observed enrichment of liver DEGs in immune response related pathways such as “Leukocyte transendothelial migration”, “NOD-like receptor signaling pathway”, “Toll-like receptor signaling pathway” and “IL-17 signaling pathway”, which have been proven to play important roles in immune response (59–62). Additionally, our analysis showed that chemokines *Cxcl1* and *Cxcl2* were differentially regulated in the liver of the DSS group. *Cxcl1* and *Cxcl2* play critical roles in a variety of diseases by mediating the inflammatory response, and their expression rise significantly during this process, which was in accordance with our results (63, 64). Except for the enrichment of liver DEGs in immune response-related pathways, the downregulation was observed in lipid metabolism pathways, including “Fatty acid degradation”, “Fatty acid elongation” and “PPAR signaling pathway”, as well as the upregulation of “Regulation of lipolysis in adipocytes” signaling pathway (**Supplementary File 1**). The changes in these pathways suggested a reduced ability of the liver to utilize lipids and an increased lipolysis, which would cause lipid accumulation in the serum (65). In brief, The dysfunctional lipid metabolism associated with DSS-induced colitis might be due to an overall breakdown of metabolic function (51, 66). *Elovl3* is a gene involved in the synthesis of saturated and monounsaturated long-chain fatty acids, its down-regulated expression in the DSS group suggested that lipid synthesis in the liver was impaired, which also indicated a certain degree of liver injury (67). In the colon, the immune response-related pathways like were significantly enriched. Among them, MAPK signaling pathway is reported to be involved in various physiological processes including inflammation, apoptosis, stress, cell proliferation and differentiation (68). Activation of MAPK signaling pathway was also observed in DSS mice (69). Interestingly, consistent with the liver results, *Cxcl1* and *Cxcl2* gene expression, which were enriched in the IL-17 signaling pathway, were significantly up-regulated. Kong et al. (26) reported that DEGs in the colon of DSS-treated mice were involved in inflammatory response, immune response, etc. These results further supported that severe inflammation occurred in the intestine of DSS-treated mice. Intestinal damage was also intimately linked to the downregulation of “DNA replication” and “Cell cycle” pathways. The downregulation of these two pathways often lead to cell damage, tumors, cancer and other diseases (70, 71). Based on these results, we hypothesized that DSS-induced colitis and secondary liver injury could be mainly mediated through modulation of immune response and lipid metabolism pathways, while intestinal damage could also be associated with DNA damage repair and cell growth and death. These results also suggested that the alleviation of lipid metabolism disorders might be one of the entry points for the future treatment of colitis, which provides new insights for the subsequent development of novel therapeutic agents. However, the precise mechanism of DSS-induced disruption of lipid metabolism in mice with colitis needs to be further explored.

Apart from host gene regulation, several studies have shown that the gut microbiota plays an important role in maintaining gut homeostasis and in liver health through the liver-microbiome axis (15, 72). The gut microbiota of patients with IBD is different from that of healthy individuals (73). DSS-induced colitis is also accompanied by strong microbiome shifts, such as dysbiosis expansion of the harmful bacteria *Escherichia-Shigella* (74), and it has been reported that the abundance of *Escherichia-Shigella* is significantly increased in the stool of colitis patients (75, 76). Consistently, in our study, the structure and composition of the colonic microbiome after DSS treatment was distinctly different from that of the CON group, with a notably elevated relative abundance of harmful bacteria and a significantly decreased relative abundance of beneficial bacteria in the DSS group, which indicated that DSS-induced gut microbiota dysbiosis might be one of the causes of exacerbating intestinal and liver injury.

As previously reported, the intestinal microbiome is strongly interacting with host gene expression (77). Therefore, its superficial mechanisms were further parsed in this work from the perspective of gut microbe -host gene interactions. In our study, we observed some correlations between intestinal microbes and DEGs in liver and colon. Consistent with previous reports, the relative abundance of *Escherichia_Shigella* and *Butyricimonas* was higher in colitis mice and lower in *Faecalibaculum* and *Eisenbergiella*, which favor intestinal health (78, 79). *Escherichia_Shigella* had the highest correlation with *Lepr*, which has been reported to be involved in the process of liver injury and inflammatory response (80, 81). The highest positive correlation with *Faecalibaculum* was found with *Etnppl*, a gene downregulated in hepatocellular carcinoma and lipid metabolism disorders (82). *Cyp4a12b*, a gene belonging to the cytochrome P450 system that was highly negatively associated with *Escherichia_Shigella*, has been reported to be downregulated in the liver and intestine of DSS-induced mice (83). Interestingly, *Escherichia_Shigella* played a primary role in both the immune response and lipid metabolism pathways in the colon. This finding was also supported by previous studies that the abundance of *Escherichia_Shigella* is higher in mice with DSS-induced colitis and patients with ulcerative colitis than in healthy individuals (84, 85). This suggested that *Escherichia_Shigella* might be one of the key strains causing colitis. *Cntnap2* was the gene with the most positive relevance to *Escherichia_Shigella*, and although this gene has not been studied in colitis, Buffington et al. (86) reported that *Cntnap2* interacts with the gut microbiota. *Myb* has been reported to be downregulated in patients with ulcerative colitis (87). Therefore, we speculated that the gut microbiota might be involved in regulating the expression of genes like *Lepr*, *Etnppl*, and *Myb* through their metabolites, which in turn drive further intestinal and liver injury. However, the precise mechanism needs to be investigated in depth to explore the role of gut microbe -host gene interactions in DSS-induced colitis and secondary liver injury.

In summary, the results obtained in this study indicated the risk of liver injury in DSS-induced colitis. This may be related to changes in the composition and structure of the gut microbiota during the development of colitis, especially the increased relative abundance of harmful bacteria such as *Escherichia_Shigella*, which led to dysbiosis of the gut bacteria and the production of harmful metabolites. Subsequently, the dysregulated gut bacteria further impaired intestinal and liver health *via* affecting the expression of genes (e.g., *Lepr, Cxcl1, Elovl3, Cyp4a12b, Myb*) enriched in the immune response, lipid metabolism and damage repair pathways. As for how gut bacteria affect gene expression in the host, it needs to be further explored. In addition, our data furnish candidate interactions between colonic microbiota of colitis mice and genes in the colon and liver, revealing the effects of host-microbiota interactions and their correlation with liver injury and colitis in DSS-induce mice. Nevertheless, this study mainly focused on the correlations of colonic microbiota and host gene expression profiles to investigate the liver injury and its underlying mechanisms caused by DSS-induced colitis, the exact causality and precise mechanisms still need to be clarified by more detailed and comprehensive studies. It should also be pointed out that the DSS-induced colitis model is unable to realistically reproduce the features of real human colitis. The limitations should consistently be taken into account when applying laboratory data directly to the human disease.

## Data Availability Statement

The original contributions presented in the study are publicly available. This data can be found here: https://www.ncbi.nlm.nih.gov/bioproject/PRJNA753214, https://www.ncbi.nlm.nih.gov/geo/query/acc.cgi?acc=GSE181959.

## Ethics Statement

The animal study was reviewed and approved by Animal Care and Use Committee and the Animal Experimentation Ethics Committee of Zhejiang University.

## Author Contributions

LZ and XZ, data acquisition and analysis and writing original manuscripts. XX, data analysis. YC and JF, methodology. ZL and MJ, revising the work critically for important intellectual content. FW, reviewing the manuscript. XZ and YW, the conception and design of the study, supervision, funding acquisition, final approval of the version to be submitted. All authors contributed to the article and approved the submitted version.

## Funding

This work was funded by the Zhejiang Provincial Key R&D Program of China (2021C02008), National Natural Science Foundation of China (Grant No. 32002185 and 31630075).

## Conflict of Interest

The authors declare that the research was conducted in the absence of any commercial or financial relationships that could be construed as a potential conflict of interest.

## Publisher’s Note

All claims expressed in this article are solely those of the authors and do not necessarily represent those of their affiliated organizations, or those of the publisher, the editors and the reviewers. Any product that may be evaluated in this article, or claim that may be made by its manufacturer, is not guaranteed or endorsed by the publisher.

## References

[1] Zhang YZ Li YY. Inflammatory Bowel Disease: Pathogenesis. World J Gastroenterol (2014) 20:91–9. doi: 10.3748/wjg.v20.i1.91 PMC3886036 24415861

[2] Kaplan GG. The Global Burden of IBD: From 2015 to 2025. Nat Rev Gastroenterol Hepatol (2015) 12:720–7. doi: 10.1038/nrgastro.2015.150 26323879

[3] Zezos P Kouklakis G Saibil F. Inflammatory Bowel Disease and Thromboembolism. World J Gastroenterol (2014) 20:13863–78. doi: 10.3748/wjg.v20.i38.13863 PMC4194568 25320522

[4] Rieder F Zimmermann EM Remzi FH Sandborn WJ. Crohn's Disease Complicated by Strictures: A Systematic Review. Gut (2013) 62:1072–84. doi: 10.1136/gutjnl-2012-304353 PMC4884453 23626373

[5] Zuo T Ng SC. The Gut Microbiota in the Pathogenesis and Therapeutics of Inflammatory Bowel Disease. Front Microbiol (2018) 9:2247. doi: 10.3389/fmicb.2018.02247 30319571 PMC6167487

[6] McGovern DP Kugathasan S Cho JH. Genetics of Inflammatory Bowel Diseases. Gastroenterology (2015) 149:1163–1176.e1162. doi: 10.1053/j.gastro.2015.08.001 26255561 PMC4915781

[7] Piovani D Danese S Peyrin-Biroulet L Nikolopoulos GK Lytras T Bonovas S. Environmental Risk Factors for Inflammatory Bowel Diseases: An Umbrella Review of Meta-Analyses. Gastroenterology (2019) 157:647–659.e644. doi: 10.1053/j.gastro.2019.04.016 31014995

[8] Kim SH Lee W Kwon D Lee S Son SW Seo MS. Metabolomic Analysis of the Liver of a Dextran Sodium Sulfate-Induced Acute Colitis Mouse Model: Implications of the Gut-Liver Connection. Cells (2020) 9:341. doi: 10.3390/cells9020341 PMC7072179 32024178

[9] Yu Y Blokhuis BR Garssen J. A Transcriptomic Insight Into the Impact of Colon Cancer Cells on Mast Cells. Int J Mol Sci (2019) 20:1689. doi: 10.3390/ijms20071689 PMC6480031 30987352

[10] Verheijen J Sleegers K. Understanding Alzheimer Disease at the Interface Between Genetics and Transcriptomics. Trends Genet (2018) 34:434–47. doi: 10.1016/j.tig.2018.02.007 29573818

[11] Zhao S. Alternative Splicing, RNA-Seq and Drug Discovery. Drug Discov Today (2019) 24:1258–67. doi: 10.1016/j.drudis.2019.03.030 30953866

[12] Xavier RJ Podolsky DK. Unravelling the Pathogenesis of Inflammatory Bowel Disease. Nature (2007) 448:427–34. doi: 10.1038/nature06005 17653185

[13] Marshall JC. The Gut as a Potential Trigger of Exercise-Induced Inflammatory Responses. Can J Physiol Pharmacol (1998) 76:479–84. doi: 10.1139/cjpp-76-5-479 9839072

[14] Albillos A de Gottardi A Rescigno M. The Gut-Liver Axis in Liver Disease: Pathophysiological Basis for Therapy. J Hepatol (2020) 72:558–77. doi: 10.1016/j.jhep.2019.10.003 31622696

[15] Lin L Zhang J. Role of Intestinal Microbiota and Metabolites on Gut Homeostasis and Human Diseases. BMC Immunol (2017) 18:2. doi: 10.1186/s12865-016-0187-3 28061847 PMC5219689

[16] Song M Chan AT. Environmental Factors, Gut Microbiota, and Colorectal Cancer Prevention. Clin Gastroenterol Hepatol (2019) 17:275–89. doi: 10.1016/j.cgh.2018.07.012 PMC6314893 30031175

[17] Zong X Fu J Xu B Wang Y Jin M. Interplay Between Gut Microbiota and Antimicrobial Peptides. Anim Nutr (2020) 6:389–96. doi: 10.1016/j.aninu.2020.09.002 PMC7750803 33364454

[18] Wang K Jin X Li Q Sawaya A Le Leu RK Conlon MA. Propolis From Different Geographic Origins Decreases Intestinal Inflammation and Bacteroides Spp. Populations in a Model of DSS-Induced Colitis. Mol Nutr Food Res (2018) 62:e1800080. doi: 10.1002/mnfr.201800080 29889351

[19] Chen L Li J Ye Z Sun B Wang L Chen Y. Anti-High Mobility Group Box 1 Neutralizing-Antibody Ameliorates Dextran Sodium Sulfate Colitis in Mice. Front Immunol (2020) 11:585094. doi: 10.3389/fimmu.2020.585094 33193406 PMC7661783

[20] Loher F Schmall K Freytag P Landauer N Hallwachs R Bauer C. The Specific Type-4 Phosphodiesterase Inhibitor Mesopram Alleviates Experimental Colitis in Mice. J Pharmacol Exp Ther (2003) 305:549–56. doi: 10.1124/jpet.102.039529 12606674

[21] Zong X Cao X Wang H Xiao X Wang Y Lu Z. Cathelicidin-WA Facilitated Intestinal Fatty Acid Absorption Through Enhancing PPAR-γ Dependent Barrier Function. Front Immunol (2019) 10:1674. doi: 10.3389/fimmu.2019.01674 31379865 PMC6650583

[22] Zong X Xiao X Shen B Jiang Q Wang H Lu Z. The N6-Methyladenosine RNA-Binding Protein YTHDF1 Modulates the Translation of TRAF6 to Mediate the Intestinal Immune Response. Nucleic Acids Res (2021) 49:5537–52. doi: 10.1093/nar/gkab343 PMC8191762 33999206

[23] Lozupone C Knight R. UniFrac: A New Phylogenetic Method for Comparing Microbial Communities. Appl Environ Microbiol (2005) 71:8228–35. doi: 10.1128/aem.71.12.8228-8235.2005 PMC1317376 16332807

[24] Segata N Izard J Waldron L Gevers D Miropolsky L Garrett WS. Metagenomic Biomarker Discovery and Explanation. Genome Biol (2011) 12:R60. doi: 10.1186/gb-2011-12-6-r60 21702898 PMC3218848

[25] Douglas GM Maffei VJ. PICRUSt2 for Prediction of Metagenome Functions. Nat Biotechnol (2020) 38:685–8. doi: 10.1038/s41587-020-0548-6 PMC7365738 32483366

[26] Kong C Yan X Liu Y Huang L Zhu Y He J. Ketogenic Diet Alleviates Colitis by Reduction of Colonic Group 3 Innate Lymphoid Cells Through Altering Gut Microbiome. Signal Transduct Target Ther (2021) 6:154. doi: 10.1038/s41392-021-00549-9 33888680 PMC8062677

[27] Love MI Huber W Anders S. Moderated Estimation of Fold Change and Dispersion for RNA-Seq Data With Deseq2. Genome Biol (2014) 15:550. doi: 10.1186/s13059-014-0550-8 25516281 PMC4302049

[28] Conesa A Madrigal P Tarazona S Gomez-Cabrero D Cervera A McPherson A. A Survey of Best Practices for RNA-Seq Data Analysis. Genome Biol (2016) 17:13. doi: 10.1186/s13059-016-0881-8 26813401 PMC4728800

[29] Hart SN Therneau TM Zhang Y Poland GA Kocher JP. Calculating Sample Size Estimates for RNA Sequencing Data. J Comput Biol (2013) 20:970–8. doi: 10.1089/cmb.2012.0283 PMC3842884 23961961

[30] Elson CO Sartor RB Tennyson GS Riddell RH. Experimental Models of Inflammatory Bowel Disease. Gastroenterology (1995) 109:1344–67. doi: 10.1016/0016-5085(95)90599-5 7557106

[31] Yan Y Kolachala V Dalmasso G Nguyen H Laroui H Sitaraman SV. Temporal and Spatial Analysis of Clinical and Molecular Parameters in Dextran Sodium Sulfate Induced Colitis. PloS One (2009) 4:e6073. doi: 10.1371/journal.pone.0006073 19562033 PMC2698136

[32] Mahfouz M Martin P Carrion AF. Hepatic Complications of Inflammatory Bowel Disease. Clin Liver Dis (2019) 23:191–208. doi: 10.1016/j.cld.2018.12.003 30947871

[33] Ding HR Wang JL Ren HZ. Lipometabolism and Glycometabolism in Liver Diseases. Biomed Res Int (2018) 2018:1287127. doi: 10.1155/2018/1287127 31205932 PMC6530156

[34] Duan S Du X Chen S Liang J Huang S Hou S. Effect of Vitexin on Alleviating Liver Inflammation in a Dextran Sulfate Sodium (DSS)-Induced Colitis Model. BioMed Pharmacother (2020) 121:109683. doi: 10.1016/j.biopha.2019.109683 31810123

[35] De Filippo K Dudeck A Hasenberg M Nye E van Rooijen N Hartmann K. Mast Cell and Macrophage Chemokines CXCL1/CXCL2 Control the Early Stage of Neutrophil Recruitment During Tissue Inflammation. Blood (2013) 121:4930–7. doi: 10.1182/blood-2013-02-486217 23645836

[36] Westerberg R Månsson JE Golozoubova V Shabalina IG Backlund EC Tvrdik P. ELOVL3 is an Important Component for Early Onset of Lipid Recruitment in Brown Adipose Tissue. J Biol Chem (2006) 281:4958–68. doi: 10.1074/jbc.M511588200 16326704

[37] Nicoletti A Ponziani FR Biolato M Valenza V Marrone G Sganga G. Intestinal Permeability in the Pathogenesis of Liver Damage: From non-Alcoholic Fatty Liver Disease to Liver Transplantation. World J Gastroenterol (2019) 25:4814–34. doi: 10.3748/wjg.v25.i33.4814 PMC6737313 31543676

[38] Sommer F Anderson JM Bharti R Raes J Rosenstiel P. The Resilience of the Intestinal Microbiota Influences Health and Disease. Nat Rev Microbiol (2017) 15:630–8. doi: 10.1038/nrmicro.2017.58 28626231

[39] Shen ZH Zhu CX Quan YS Yang ZY Wu S Luo WW. Relationship Between Intestinal Microbiota and Ulcerative Colitis: Mechanisms and Clinical Application of Probiotics and Fecal Microbiota Transplantation. World J Gastroenterol (2018) 24:5–14. doi: 10.3748/wjg.v24.i1.5 29358877 PMC5757125

[40] Motoi Y Ito Z Suzuki S Takami S Matsuo K Sato M. FADS2 and ELOVL6 Mutation Frequencies in Japanese Crohn's Disease Patients. Drug Discov Ther (2019) 13:354–9. doi: 10.5582/ddt.2019.01081 31956234

[41] Ng SC Shi HY Hamidi N Underwood FE Tang W Benchimol EI. Worldwide Incidence and Prevalence of Inflammatory Bowel Disease in the 21st Century: A Systematic Review of Population-Based Studies. Lancet (2017) 390:2769–78. doi: 10.1016/s0140-6736(17)32448-0 29050646

[42] Molodecky NA Soon IS Rabi DM Ghali WA Ferris M Chernoff G. Increasing Incidence and Prevalence of the Inflammatory Bowel Diseases With Time, Based on Systematic Review. Gastroenterology (2012) 142:46–54.e42; quiz e30. doi: 10.1053/j.gastro.2011.10.001 22001864

[43] Gao X Cao Q Cheng Y Zhao D Wang Z Yang H. Chronic Stress Promotes Colitis by Disturbing the Gut Microbiota and Triggering Immune System Response. Proc Natl Acad Sci U S A (2018) 115:E2960–9. doi: 10.1073/pnas.1720696115 29531080 PMC5879702

[44] Bauer C Duewell P Mayer C Lehr HA Fitzgerald KA Dauer M. Colitis Induced in Mice With Dextran Sulfate Sodium (DSS) is Mediated by the NLRP3 Inflammasome. Gut (2010) 59:1192–9. doi: 10.1136/gut.2009.197822 20442201

[45] Wang Z Hao C Zhuang Q Zhan B Sun X Huang J. Excretory/Secretory Products From Trichinella Spiralis Adult Worms Attenuated DSS-Induced Colitis in Mice by Driving PD-1-Mediated M2 Macrophage Polarization. Front Immunol (2020) 11:563784. doi: 10.3389/fimmu.2020.563784 33117347 PMC7575908

[46] Xiao YT Yan WH Cao Y Yan JK Cai W. Neutralization of IL-6 and TNF-α Ameliorates Intestinal Permeability in DSS-Induced Colitis. Cytokine (2016) 83:189–92. doi: 10.1016/j.cyto.2016.04.012 27155817

[47] Singh UP Singh NP Murphy EA Price RL Fayad R Nagarkatti M. Chemokine and Cytokine Levels in Inflammatory Bowel Disease Patients. Cytokine (2016) 77:44–9. doi: 10.1016/j.cyto.2015.10.008 PMC4666758 26520877

[48] Gupta A Yu A Peyrin-Biroulet L Ananthakrishnan AN. Treat to Target: The Role of Histologic Healing in Inflammatory Bowel Diseases: A Systematic Review and Meta-Analysis. Clin Gastroenterol Hepatol (2020) 19:1800–13.e4. doi: 10.1016/j.cgh.2020.09.046 33010406

[49] Sharma D Malik A Guy CS Karki R Vogel P Kanneganti TD. Pyrin Inflammasome Regulates Tight Junction Integrity to Restrict Colitis and Tumorigenesis. Gastroenterology (2018) 154:948–964.e948. doi: 10.1053/j.gastro.2017.11.276 29203393 PMC5847456

[50] Rtibi K Grami D Wannes D Selmi S Amri M Sebai H. Ficus Carica Aqueous Extract Alleviates Delayed Gastric Emptying and Recovers Ulcerative Colitis-Enhanced Acute Functional Gastrointestinal Disorders in Rats. J Ethnopharmacol (2018) 224:242–9. doi: 10.1016/j.jep.2018.06.001 29870788

[51] Zheng H Wang J Wei X Chang L Liu S. Proinflammatory Properties and Lipid Disturbance of Polystyrene Microplastics in the Livers of Mice With Acute Colitis. Sci Total Environ (2021) 750:143085. doi: 10.1016/j.scitotenv.2020.143085 33182181

[52] Agouridis AP Elisaf M Milionis HJ. An Overview of Lipid Abnormalities in Patients With Inflammatory Bowel Disease. Ann Gastroenterol (2011) 24:181–7. PMC3959314 24713706

[53] Trefts E Gannon M Wasserman DH. The Liver. Curr Biol (2017) 27:R1147–r1151. doi: 10.1016/j.cub.2017.09.019 29112863 PMC5897118

[54] Ooi K Shiraki K Sakurai Y Morishita Y Nobori T. Clinical Significance of Abnormal Lipoprotein Patterns in Liver Diseases. Int J Mol Med (2005) 15:655–60. doi: 10.3892/ijmm.15.4.655 15754028

[55] Rojas-Feria M Castro M Suárez E Ampuero J Romero-Gómez M. Hepatobiliary Manifestations in Inflammatory Bowel Disease: The Gut, the Drugs and the Liver. World J Gastroenterol (2013) 19:7327–40. doi: 10.3748/wjg.v19.i42.7327 PMC3831215 24259964

[56] Thin LW Lawrance IC Spilsbury K Kava J Olynyk JK. Detection of Liver Injury in IBD Using Transient Elastography. J Crohns Colitis (2014) 8:671–7. doi: 10.1016/j.crohns.2013.12.006 24529605

[57] Zheng C Zheng L Yoo JK Guo H Zhang Y Guo X. Landscape of Infiltrating T Cells in Liver Cancer Revealed by Single-Cell Sequencing. Cell (2017) 169:1342–1356.e1316. doi: 10.1016/j.cell.2017.05.035 28622514

[58] Hansen KD Wu Z Irizarry RA Leek JT. Sequencing Technology Does Not Eliminate Biological Variability. Nat Biotechnol (2011) 29:572–3. doi: 10.1038/nbt.1910 PMC3137276 21747377

[59] Getter T Margalit R Kahremany S Levy L Blum E Khazanov N. Novel Inhibitors of Leukocyte Transendothelial Migration. Bioorg Chem (2019) 92:103250. doi: 10.1016/j.bioorg.2019.103250 31580982

[60] Platnich JM Muruve DA. NOD-Like Receptors and Inflammasomes: A Review of Their Canonical and non-Canonical Signaling Pathways. Arch Biochem Biophys (2019) 670:4–14. doi: 10.1016/j.abb.2019.02.008 30772258

[61] Takeda K Akira S. TLR Signaling Pathways. Semin Immunol (2004) 16:3–9. doi: 10.1016/j.smim.2003.10.003 14751757

[62] McGeachy MJ Cua DJ Gaffen SL. The IL-17 Family of Cytokines in Health and Disease. Immunity (2019) 50:892–906. doi: 10.1016/j.immuni.2019.03.021 30995505 PMC6474359

[63] Kobayashi Y. The Role of Chemokines in Neutrophil Biology. Front Biosci (2008) 13:2400–7. doi: 10.2741/2853 17981721

[64] Volzke J Schultz D Kordt M Müller M Bergmann W Methling K. Inflammatory Joint Disease Is a Risk Factor for Streptococcal Sepsis and Septic Arthritis in Mice. Front Immunol (2020) 11:579475. doi: 10.3389/fimmu.2020.579475 33117382 PMC7576673

[65] Mao Z Feng M Li Z Zhou M Xu L Pan K. ETV5 Regulates Hepatic Fatty Acid Metabolism Through PPAR Signaling Pathway. Diabetes (2021) 70:214–26. doi: 10.2337/db20-0619 33093014

[66] Kwon J Lee C Heo S Kim B Hyun CK. DSS-Induced Colitis is Associated With Adipose Tissue Dysfunction and Disrupted Hepatic Lipid Metabolism Leading to Hepatosteatosis and Dyslipidemia in Mice. Sci Rep (2021) 11:5283. doi: 10.1038/s41598-021-84761-1 33674694 PMC7935975

[67] Zadravec D Brolinson A Fisher RM Carneheim C Csikasz RI Bertrand-Michel J. Ablation of the Very-Long-Chain Fatty Acid Elongase ELOVL3 in Mice Leads to Constrained Lipid Storage and Resistance to Diet-Induced Obesity. FASEB J (2010) 24:4366–77. doi: 10.1096/fj.09-152298 20605947

[68] Johnson GL Lapadat R. Mitogen-Activated Protein Kinase Pathways Mediated by ERK, JNK, and P38 Protein Kinases. Science (2002) 298:1911–2. doi: 10.1126/science.1072682 12471242

[69] Zhang Z Liu J Shen P Cao Y Lu X Gao X. Zanthoxylum Bungeanum Pericarp Extract Prevents Dextran Sulfate Sodium-Induced Experimental Colitis in Mice *via* the Regulation of TLR4 and TLR4-Related Signaling Pathways. Int Immunopharmacol (2016) 41:127–35. doi: 10.1016/j.intimp.2016.10.021 27843005

[70] Lam FC Kong YW Huang Q Vu Han TL. BRD4 Prevents the Accumulation of R-Loops and Protects Against Transcription-Replication Collision Events and DNA Damage. Nat Commun (2020) 11:4083. doi: 10.1038/s41467-020-17503-y 32796829 PMC7428008

[71] Russo M Crisafulli G Sogari A Reilly NM Bardelli A. Adaptive Mutability of Colorectal Cancers in Response to Targeted Therapies. Science (2019) 366:1473–80. doi: 10.1126/science.aav4474 31699882

[72] Adolph TE Grander C Moschen AR Tilg H. Liver-Microbiome Axis in Health and Disease. Trends Immunol (2018) 39:712–23. doi: 10.1016/j.it.2018.05.002 29843959

[73] Glassner KL Abraham BP Quigley EMM. The Microbiome and Inflammatory Bowel Disease. J Allergy Clin Immunol (2020) 145:16–27. doi: 10.1016/j.jaci.2019.11.003 31910984

[74] Cevallos SA Lee JY Velazquez EM Foegeding NJ Shelton CD Tiffany CR. 5-Aminosalicylic Acid Ameliorates Colitis and Checks Dysbiotic Escherichia Coli Expansion by Activating PPAR-γ Signaling in the Intestinal Epithelium. mBio (2021) 12:e03227–20. doi: 10.1128/mBio.03227-20 PMC7845635 33468700

[75] Kim ES Tarassishin L Eisele C Barre A Nair N Rendon A. Longitudinal Changes in Fecal Calprotectin Levels Among Pregnant Women With and Without Inflammatory Bowel Disease and Their Babies. Gastroenterology (2021) 160:1118–1130.e1113. doi: 10.1053/j.gastro.2020.11.050 33307026

[76] He XX Li YH Yan PG Meng XC Chen CY Li KM. Relationship Between Clinical Features and Intestinal Microbiota in Chinese Patients With Ulcerative Colitis. World J Gastroenterol (2021) 27:4722–37. doi: 10.3748/wjg.v27.i28.4722 PMC8326252 34366632

[77] Dayama G Priya S Niccum DE Khoruts A Blekhman R. Interactions Between the Gut Microbiome and Host Gene Regulation in Cystic Fibrosis. Genome Med (2020) 12:12. doi: 10.1186/s13073-020-0710-2 31992345 PMC6988342

[78] Zagato E Pozzi C. Endogenous Murine Microbiota Member Faecalibaculum Rodentium and its Human Homologue Protect From Intestinal Tumour Growth. Nat Microbiol (2020) 5:511–24. doi: 10.1038/s41564-019-0649-5 PMC7048616 31988379

[79] Li X Brejnrod AD Ernst M Rykær M Herschend J Olsen NMC. Heavy Metal Exposure Causes Changes in the Metabolic Health-Associated Gut Microbiome and Metabolites. Environ Int (2019) 126:454–67. doi: 10.1016/j.envint.2019.02.048 30844581

[80] Vuletic MS Milosevic VS Jancic SA Zujovic JT Krstic MS Vukmirovic FC. Clinical Significance of Leptin Receptor (LEPR) and Endoglin (CD105) Expressions in Colorectal Adenocarcinoma. J BUON (2019) 24:2448–57. 31983119

[81] Zhao Z Peng W Zhou J Zhou Y Liu T Bai H. Association of LEPR Polymorphisms With Predisposition and Inflammatory Response in Anti-Tuberculosis Drug-Induced Liver Injury: A Pilot Prospective Investigation in Western Chinese Han Population. Infect Genet Evol (2019) 75:103970. doi: 10.1016/j.meegid.2019.103970 31325611

[82] Ding Q Kang J Dai J Tang M Wang Q Zhang H. AGXT2L1 is Down-Regulated in Heptocellular Carcinoma and Associated With Abnormal Lipogenesis. J Clin Pathol (2016) 69:215–20. doi: 10.1136/jclinpath-2015-203042 26294768

[83] Fan X Ding X Zhang QY. Hepatic and Intestinal Biotransformation Gene Expression and Drug Disposition in a Dextran Sulfate Sodium-Induced Colitis Mouse Model. Acta Pharm Sin B (2020) 10:123–35. doi: 10.1016/j.apsb.2019.12.002 PMC6976992 31993311

[84] Jialing L Yangyang G Jing Z Xiaoyi T Ping W Liwei S. Changes in Serum Inflammatory Cytokine Levels and Intestinal Flora in a Self-Healing Dextran Sodium Sulfate-Induced Ulcerative Colitis Murine Model. Life Sci (2020) 263:118587. doi: 10.1016/j.lfs.2020.118587 33065145

[85] Burke D. Escherichia Coli and Ulcerative Colitis. J R Soc Med (1997) 90:612–7. doi: 10.1177/014107689709001106 PMC1296671 9496273

[86] Buffington SA Dooling SW Sgritta M Noecker C Murillo OD Felice DF. Dissecting the Contribution of Host Genetics and the Microbiome in Complex Behaviors. Cell (2021) 184:1740–1756.e1716. doi: 10.1016/j.cell.2021.02.009 33705688 PMC8996745

[87] Bian Z Li L Cui J Zhang H Liu Y Zhang CY. Role of miR-150-Targeting C-Myb in Colonic Epithelial Disruption During Dextran Sulphate Sodium-Induced Murine Experimental Colitis and Human Ulcerative Colitis. J Pathol (2011) 225:544–53. doi: 10.1002/path.2907 21590770

